# Athletics and Albuminuria

**Published:** 1907-07-13

**Authors:** 


### Clinical Points.

### ATHLETICS AND ALBUMINURIA.

The question of the significance of the albuminuria which is so often found in young males over puberty, and which is called by various names such as "functional," "physiological," "postural," "cyclic," and so on, is one upon which views have greatly changed during the last ten years. There are two large classes who still suffer from the effects of older views, namely, young men who wish to insure their lives, and young men who are desirous of taking posts in one or other of the public services, in banks, and similar places.

The older view was that this albuminuria of adolescent manhood was a sign of evil omen, and that it indicated an instability of the kidneys, which, if not actually the seat of Bright's disease, were at least prone to it. There are, of course, cases of actual Bright's disease in young men; there are cases of albuminuria from heart disease, from blood diseases, and so forth; it is not to these that the term functional albuminuria applies, but to those youths and young men who may never have had a day's illness since they were children, who feel and look well, and who come up for medical examination in the full expectation of being passed. We mean, moreover, those in whose urine the presence of albumen in the urine is not constant, large amounts being found at one time and none at all at another, while microscopical examination of the deposit discovers no pathological number of renal tube casts. We say "pathological number," because urines which are centrifugalised to the extent they are nowadays in clinical laboratories will very often be found to yield a cast or two, even when there is no albumen in the urine, and there is every reason to suppose that the individual who passed the urine is absolutely sound and well.

Every doctor who has had any number of boys at school to examine, or who has examined candidates for life insurance, or for bank-clerkships and the like, has come across the difficulty of knowing what to advise when a hopeful candidate is found to have this albuminuria. In the olden days the candidate would have been rejected forthwith as an unsafe life; at present it is the common rule at insurance offices to postpone the proposal on the life for some months, during which time several separate examinations of the urine are made at intervals; if albuminuria is found every time, the proposal is declined; if albumen is present at some examinations and not at others, the risk upon the life may be accepted with a load of from five to seven or ten years.

We feel sure, however, that this constitutes a hardship upon those insured; for, as we shall see presently, the prognosis in these "functional" cases is as good as it is in a person of the same age without albumen in his urine. We think it quite essential that the insurance offices should safeguard themselves against accepting cases of actual nephritis, by postponing the acceptance of the proposal until further examinations of the urine have shown that it is sometimes quite free from albumen; candidates would be well advised to have an early morning specimen examined if they wish to get one free from albumen, because the more walking about and exercise there has been before the urine was passed, the greater is the likelihood of its containing albumen. We even think that, granted that the urine is sometimes free from albumen, the companies should still insist upon having a microscopical examination of the centrifugalised deposit carried out by a skilled person, to be sure that no pus cells, blood corpuscles, nor renal tube casts are present. When, however, both the absence of albumen upon occasions and the absence of pathological findings under the microscope have proved that the condition in any particular case is "functional," then we think that no load is necessary in accepting the life; we believe that the office which would accept these lives at tabular rates would find them good, and that the time is coming when these lives will not be loaded.

The evidence upon which this view is based is derived from numerous papers upon the subject; perhaps the most convincing of these is one by Dr. Collier, based upon examinations of the urines of undergraduates at Oxford, before and after rowing or other exercises. Dr. Collier found that, almost without exception, every rowing man had abundance of albumen in his urine after rowing a race, while the same man before the race, or a few hours afterwards, when he had changed his clothes and rested, had no albuminuria at all. Dr. Collier says: "I could produce several young men at Oxford who, if they wished to insure themselves or enter for the Indian Civil Service, would be accepted as first-class lives if they were examined in the morning, and would fail in the afternoon, because after a certain amount of exercise their urine would show a definite amount of albumen." Dr. Dunhill previously published statistics precisely similar to those of Dr. Collier; in every single member of the crew at Melbourne University he found albumen in abundance after a race, though there was no albuminuria at other times in the same men. Urine was collected from eight competitors in a ten-mile cross country run immediately after they had finished; every one of the specimens contained albumen.

It is clear, therefore, that severe bodily exercise not only may cause albuminuria in young males, but practically always does so. This being so, it is not at all surprising that in many young males fairly ordinary exercise will cause it. Such "functional" albuminuria is normal in these cases, differing only in degree from the normal albuminuria which occurs in all young men after severe exercise.

It will naturally be asked: What is the ultimate result in these young men who have gone in for athletics so constantly that they must have again and again had albuminuria? The answer to this has already been worked out; for it has long been known that the average length of life of the men who have gone in for rowing at the Universities is distinctly greater than is that of the rest of the average men of the same age. The risk in accepting these lives for insurance should, therefore, be a good one. Formerly, urines of athletes were not examined for albumen; in the happy ignorance of their albuminuria, the athletes and rowing men were not bothered with the question whether or not they should go on with their various forms of exercise notwithstanding albuminuria, and obviously it did them no harm. Since then many a man has been advised to discontinue his athletics because he has been found to have albuminuria after them; the doctrine that athletic albuminuria is a contraindication to vigorous exercise must now give way again, and athletes need pay little or no heed to their albuminuria. In Dr. Collier's words:—

"Ought one any longer to advise young men who pass large quantities of albumen after severe muscular exercise to give up all hard competitions? I think not. These investigations would seem to prove that, if so, we must discourage severe athletic competitions altogether. Of this year's Oxford University crew every member after rowing a trial over the full course passed a definite amount of albumen, and at least half the crew passed a very considerable quantity. With the college crews the same thing happened. In the New College boat, head of the river in the Torpids, after rowing a course every member's urine contained some, while in five of the crew the amount was large. The running men seemed to pass even more than the rowing men. . . . Finally, ought the assurance companies to continue to refuse to consider the acceptance of the lives of young men between the ages of 18 and 30, whose urines are found to contain albumen after exercise when it can be shown that no albumen is present after rest or after a meal? I think not. I have known instances of men who have been absolutely refused because they happened to be examined in the afternoon after exercise, when they would certainly have been accepted had they been examined in the earlier part of the day. To me this seems a very unsatisfactory state of affairs."

